# Study of compartmentalization in the visna clinical form of small ruminant lentivirus infection in sheep

**DOI:** 10.1186/1746-6148-8-8

**Published:** 2012-01-26

**Authors:** Hugo Ramírez, Ramsés Reina, Luigi Bertolotti, Amaia Cenoz, Mirna-Margarita Hernández, Beatriz San Román, Idoia Glaria, Ximena de Andrés, Helena Crespo, Paula Jáuregui, Julio Benavides, Laura Polledo, Valentín Pérez, Juan F García-Marín, Sergio Rosati, Beatriz Amorena, Damián de Andrés

**Affiliations:** 1 Instituto de Agrobiotecnología, CSIC-UPNA-Gobierno de Navarra, 31192 Mutilva, Navarra, Spain; 2 Dipartimento di Produzioni Animali, Epidemiologia, Ecologia, Facoltá di Medicina Veterinaria, Universitá degli Studi di Torino, Grugliasco (TO), Italy; 3 Instituto de Ganadería de Montaña (CSIC-ULE), León, Spain; 4 Facultad de Veterinaria, Universidad de León, León, Spain; 5 Laboratorio de Virología, Genética y Biología Molecular, FESC-UNAM, Veterinary C-4, 54700 Cuautitlán Izcalli, Estado de México, Mexico; 6 Instituto de Agrobiotecnología, CSIC-UPNA-Gobierno de Navarra, Ctra Mutilva s/n, 31192 Mutilva, Navarra, Spain

**Keywords:** Compartmentalization, Visna, Small ruminant lentivirus, Spinal cord, Choroid plexus, Sheep

## Abstract

**Background:**

A central nervous system (CNS) disease outbreak caused by small ruminant lentiviruses (SRLV) has triggered interest in Spain due to the rapid onset of clinical signs and relevant production losses. In a previous study on this outbreak, the role of LTR in tropism was unclear and *env *encoded sequences, likely involved in tropism, were not investigated. This study aimed to analyze heterogeneity of SRLV Env regions - TM amino terminal and SU V4, C4 and V5 segments - in order to assess virus compartmentalization in CNS.

**Results:**

Eight Visna (neurologically) affected sheep of the outbreak were used. Of the 350 clones obtained after PCR amplification, 142 corresponded to CNS samples (spinal cord and choroid plexus) and the remaining to mammary gland, blood cells, bronchoalveolar lavage cells and/or lung. The diversity of the *env *sequences from CNS was 11.1-16.1% between animals and 0.35-11.6% within each animal, except in one animal presenting two sequence types (30% diversity) in the CNS (one grouping with those of the outbreak), indicative of CNS virus sequence heterogeneity. Outbreak sequences were of genotype A, clustering per animal and compartmentalizing in the animal tissues. No CNS specific signature patterns were found.

**Conclusions:**

Bayesian approach inferences suggested that proviruses from broncoalveolar lavage cells and peripheral blood mononuclear cells represented the common ancestors (infecting viruses) in the animal and that neuroinvasion in the outbreak involved microevolution after initial infection with an A-type strain. This study demonstrates virus compartmentalization in the CNS and other body tissues in sheep presenting the neurological form of SRLV infection.

## Background

Small ruminant lentiviruses (SRLVs) cause arthritis, mastitis, interstitial pneumonia (Maedi) and leukoencephalitis (Visna) in sheep and goats [1]. The clinical form Visna in sheep was first described in Iceland during the Visna Maedi Virus (VMV) epidemic in the first half of the last century [2]. From then, this neurologic form has been reported only sporadically [3-5]. However, in the last few years, the neurological form of SRLV disease has been diagnosed in numerous sheep of North-Western Spain, causing production losses [6]. In this outbreak, clinical signs usually appear at 1-2 years of age, but have been detected in animals as young as 4 months [7]. However, in other geographic areas of the world, Visna appears in animals over 2 years of age [2,8]. Also, in the animals affected by Visna within the outbreak, lesions frequently involve the white matter of the spinal cord [9,10] whereas in reported experimental Visna cases, lesions usually appear mainly in the periventricular areas of the brain [5].

Central nervous system (CNS) infection may occur in the early phases of sheep SRLV infection, as it occurs in human CNS with human immunodeficiency virus infections (HIV; [11]). In HIV infections, viral quasispecies change in their adaptation to certain cell types, due to differences in selective pressure that may be exerted, for example, by the immune system [12]. Consequently, the flow of genes among viral subpopulations is significantly restricted, leading to genetically distinct subpopulations and development of compartmentalization [13-15]. Hence, the genetic heterogeneity between subpopulations of the individual could be explained by both an independent microevolution and the presence of related but phylogenetically distinct infecting virus genotypes [15,16]. Viral populations distributed in compartments may have different phenotypic characteristics, such as cell tropism [17], drug resistance [18-21] and pathogenicity [22]. Further studies on viral diversification, compartmentalization and adaptation of the virus to the brain are needed [13]. The CNS provides a unique environment for the replication of lentiviruses. The blood-brain barrier presents specialized capillary endothelial cells, linked to each other by highly selective narrow bridges that may restrict viral traffic. During viral infection, a single strain appears to be able to cross the blood-brain barrier and establish the infection in the brain, which is otherwise isolated from the peripheral blood and the immune response [14,23]. Viral genome structure may also contain key features determining the fate of infection.

In SRLV infection, evidence of the relationship between SRLV-derived clinical manifestation and strain genetic features has been described [24], but there is no consensus on the specific viral genetic region that determines cell or tissue tropism [25]. Tissue tropism may be dependent on the virus promoter sequence of both VMV and HIV [26,27]. However, sequence divergence found in viral promoter does not ensure changes in transcriptional activities and/or biological characteristics. LTR alone does not exert this function and other viral genes could be involved [25].

Different HIV and feline immunodeficiency virus (FIV) studies, based on a hypervariable region of the viral surface protein (SU), have revealed tissue compartmentalization of the virus [14]. In HIV and FIV Env, the V3 region is a determinant of cell tropism and replication efficiency, since it is thought to be related to the adsorption and fusion of the virus to the cell [23,28-30]. Furthermore, in chronically infected individuals, particular amino acids of the V3 region modulate neurotropism and neurovirulence [14]. In SRLV, five variable (V1 to V5) and four conserved (C1 to C4) regions have been identified in the SU protein [31] and the function of V4 hypervariable region has been found analogous to that of the V3 region of HIV [32,33].

In this work involving Env SU regions (V4, C4 and V5), and the amino terminal sequence of the Env TM protein, we first determined the existence of viral sequence diversity in the CNS cells [choroid plexus cells (CPx), spinal cord cells (SC)], broncho-alveolar lavage macrophages (BAL), lung tissue (L), mammary gland tissue (MG), and peripheral blood mononuclear cells (PBMC) and analyzed the phylogenetic relationships of SRLV within animals belonging to an outbreak of clinical Visna [9]. Phylogeography approaches were also applied to identify the common ancestor sequences of the viral quasispecies. Finally, the existence of compartmentalization and/or presumptive positive selection of viral genomes was assessed, with special emphasis on CNS comparisons in order to better understand genetic diversity involved neurotropism and neurovirulence.

## Results

### Clinical signs and lesions

The animals included in this study presented symptoms compatible with the nervous form of VMV, such as hindleg ataxia or even paralysis, sternal or lateral recumbency and pedaling. Histopathological examination indicated the presence of severe lesions in the CNS in all the animals. In three of these animals the CNS lesions were located only in the SC, which were characterized by non-suppurative perivascular cuffs and demyelination, when affecting the neuroparenchyma. Seven of the animals showed also mild interstitial pneumonia, denoted by lymphoid infiltration in the lung alveolar walls. Only two of the animals had lesions in the MG, characteristic of a mild non-purulent interstitial mastitis (Table 1). No other pathogens were identified in the brain or other organs in the animals under study.

**Table 1:** Diagnosis and lesions. Results on PCR (LTR region), ELISA (Elitest) and histopathology obtained from eight sheep with Visna clinical symptoms belonging to the Castilla-y-León outbreak

**Animal No**.	Age (years)	Sex	Breed	LTR-PCR					Elitest ratio	Lesions				
						
				PBMC	SC	CPx	MG	L		Brain	SC	CPx	MG	L
166	2-3	M	Assaf	+	+	+	NT	+	13.87	-	+++	-	NT	+
223	> 2	F	Assaf	+	NT	+	+/-	-	18.56	+++	+++	NT	-	+
292	2	F	Assaf	-	+	-	+	+	11.06	-	+++	-	-	++
333	> 4	F	Assaf	NT	+	+	+/-	-	3.81	+++	-	+	-	+
336	3	F	Assaf	NT	+	-	+/-	-	3.37	+	+++	+	-	++
368	5	F	Milchschaf	-	+	+	+	-	5.87	+++	+++	++	-	-
697	3	F	Assaf	-	-	NT	-	-	3.12	-	+++	-	++	+
698	2	F	Assaf	+	+	-	+	-	14.56	+++	+++	+	+	+

*PBMC*, peripheral blood mononuclear cells; *SC*, spinal cord; *CPx*, choroid plexus; *MG*, mammary gland; *L*, lung; *NT*, not tested

### ELISA and PCR

All the animals had anti-SRLV antibodies as assessed by Elitest [34], with OD ratios ranging from 3 to 18 (Table 1). In spite of the availability of PBMC, BAL, MG (except for the male), L, CPx and SC samples from all the animals for PCR testing, only some samples allowed proviral amplification (Table 1). PCR amplification detected proviral LTR DNA mostly in tissues of the CNS (SC and CPx), whereas samples from L or MG yielded few positives and inconsistent results, respectively. LTR SRLV sequences obtained in this study had a classical genotype A organization and characteristics.

The *env *PCR amplification was successful in all the animals, SC being always amplified (data not shown). BAL and CPx yielded PCR positive results in 4 of the 8 animals. The number of clones was also variable among samples and animals.

### Sequence and phylogeny studies

A representative sample of the *env *amplicons was sequenced and genotyped, revealing that, like in LTR, *env *SRLV sequences obtained in this study had a classical genotype A characteristics, closer to VMV than to caprine arthritis encephalitis virus (CAEV) prototypes (not shown). Within each animal, the mean diversity of *env *sequences from CNS ranged from 0.35 to 11.6% (Table 2). The analysis of nucleotide diversity showed a quite uniform distribution of distances, except for the animal No. 333 having two distant sequence sub-sets (333a and 333b) in different tissues, including CNS. One of them grouped with those of the outbreak but not the other (30% diversity between both sequence sub-sets). Diversity observed among sequences from the CNS in different animals ranged between 11.1 and 16.1%.

**Table 2:** Nucleotide and amino acid diversity, ω statistic and ancestor tissue. Nucleotide diversity, amino acid diversity, ω statistic (ratio between average number of non-synonymous substitutions per non-synonymous site, dN; and average number of synonymous substitutions per synonymous site, dS), and ancestor tissue for SRLV clones obtained from eight sheep of the Visna outbreak

**Sheep No**.	No. of clones	Nucleotide diversity		Amino acid diversity		dN/dS (ω)	Ancestor tissue^a^
				
		Mean	Standard Error	Mean	Standard Error		
166	90	0.0542	0.0029	0.0312	0.0017	0.690547	BAL
223	32	0.0372	0.0036	0.0333	0.0039	0.982374	PBMC
292	7	0.0035	0.0007	0.0031	0.0013	0.459471	*na^b^*
333	74	0.1158	0.0104	0.0496	0.0042	0.283522	PBMC
336	64	0.0659	0.0045	0.0342	0.0026	0.442547	BAL
368	43	0.0372	0.0033	0.0312	0.0024	0.722284	BAL
697	12	0.0188	0.0043	0.0179	0.0034	0.692030	*na*
698	28	0.0178	0.0042	0.0273	0.0041	0.346625^c^	*na*

^a ^Inferences on tissue origin of most recent common ancestor were conducted only on large dataset (n > 30)

^b ^*na*: non applicable

^c ^dN/dS value (ω) was calculated excluding sequences with stop codons

**Figure 1:**
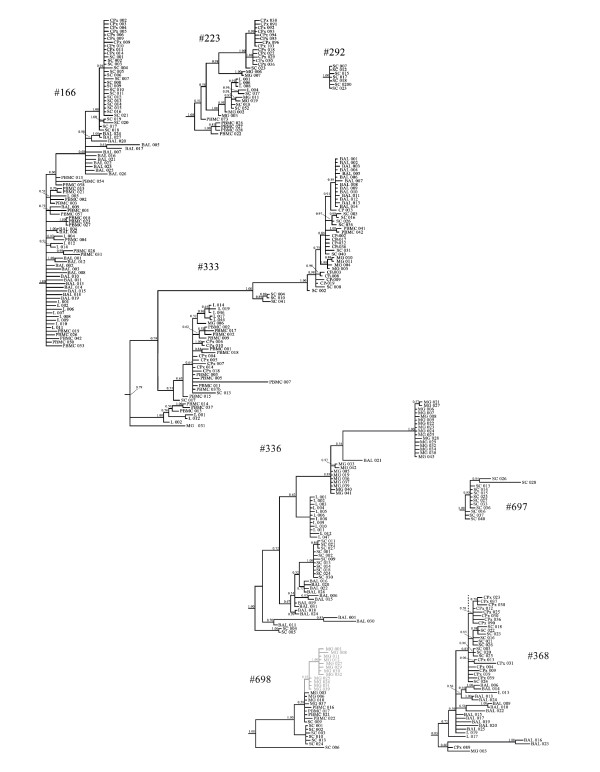
**Phylogenetic relationships among sequences belonging to the same animal**. Animal identification number is reported for each subtree. Taxa names include the tissue from which the sequence was obtained and the clone number. Tissues are coded as follow: broncho-alveolar macrophages, BAL; choroid plexus, CPx; spinal cord, SC; peripheral blood mononuclear cells, PBMC; mammary gland, MG; lung, L. Posterior probabilities of each node are reported above branches. Sequences from animal 698 with stop codons are reported in grey.

Phylogenetic analysis of SRLV sequences within animals is shown in Figure 1 and globally in Additional file 1: 'Phylogenetic tree including 350 partial *env *sequences from 8 animals'. With the exception of animal 333, sequences belonging to the same animal clearly clustered together. In addition, within the animal, sequences showed a strong tissue compartmentalization, confirming the presence of different viral sub-populations in a single host. This was compatible with either a clonal evolution after initial infection with a single strain or a polyclonal origin of infecting viral strains. Sequence distribution in tissues differed between animals. In addition, MG and L sequences frequently formed clearly defined clades, while PBMC sequences were the most variable and were usually located in different clades within an animal (Figure 1). Individual phylogram study revealed the existence of sequences in CNS, L, and/or MG that could derive from BAL and/or PBMC. In animals 166 and 368, sequences from CNS (Cpx and SC) clustered close to each other. In both animals, these sequences, when present, were also close to BAL, like SC sequences from animal 336. In fact, in sheep 166, both CNS and BAL sequences belonging to the late clade appeared to descend from BAL, L and/or PBMC (early clade). In sheep 333, there were differences in sequence distribution between the two sequence sub-sets. In one of them BAL and PBMC sequences were close to those from CNS (SC and CPx) and MG; and the other revealed the proximity of PBMC sequences to CNS (CPx), MG and L. In animal 223 CPx sequences belonged to a cluster different from that of SC sequences of the CNS, confirming the existence of different viral sequence clusters within the CNS, and PBMC sequences were close to the tree root. Also, sequences from a particular tissue appeared to have evolved within the tissue; this was the case of MG sequences in animals 336 and 698 or, less markedly SC sequences in animal 697, among others. Thus, once in the target organ, the virus seemed to have evolved, allowing the appearance of new clones within the same tissue.

The common ancestor state was then reconstructed, using Bayesian methods recently developed in phylogeography [35] (Table 2). Evolutionary parameters were estimated under the Bayesian Skyline Plot uncorrelated lognormal relaxed clock model. The convergence of chains was reached (ESS > 200 in all analyses) and Maximum Clade Credibility trees were obtained for each animal. The results showed that viral evolution within animals followed the usual infection paths. Specifically, in animals 166, 336 and 368, where sequences from BAL were present, bronchoalveolar macrophages were identified as the most likely tissue infected by the viral common ancestor. In animals 223 and 333, in which BAL samples were unavailable, PBMC had the highest probability to be the tissue infected by the viral ancestor. These data strongly suggest that the infection starts from the lungs and later reaches other tissues through the blood. An interesting aspect of these results is that CNS, lung and mammary gland are terminal clades in this evolutionary model, associated to BAL and PBMC taxa (Table 2).

In this evolutionary context, the average number of non-synonymous substitutions per non-synonymous site (dN) and the number of synonymous substitutions per synonymous site (dS) were determined for proviral sequences obtained from each animal. The dN rates were lower than the dS rates in all the animals, indicating the presence of within-host purifying selection (against amino acid sequence variability), but in favour of synonimous substitutions leading to nucleotide heterogeneity and compartmentalization (Table 2).

Compartmentalization regarding "segregation" of sequences between different animals and between tissues of a particular animal was assessed (Tables 3 and 4) according to parsimony score (PS), association index (AI) and monophyletic clade (MC) values. Results on PS and AI showed weak phylogeny-trait association and significant monophyletic clade (MC) value, altogether indicative of tissue compartmentalization in six of the studied animals. In particular, CNS sequences belonged to a different cluster compared to other tissues, within the animal. This was observed in the six animals that had at least two tissues (one of them being CNS) from which the number of sequences obtained was sufficient (≥ 28 clones per animal) to carry out the study, demonstrating and confirming the existence of CNS tissue compartmentalization. Lack of significance in PS, AI and MC analyses was observed only rarely, when a low number of sequences was available (animals 292 and 697; Table 4).

**Table 3:** SRLV sequences segregation. SRLV sequence compartmentalization (segregation) between the eight Visna-affected animals, using Bayesian MCMC approach for determination of Association Index (AI), Parsimony Score (PS) and Monophyletic Clade (MC) values

Statistic	No. of sequence	Mean value	95% Confidence interval (CI)	Significance
AI	350	0.046	1 × 10^-11 ^-0.250	*p *< 0.00001
PS	350	7.752	7-8	*p *< 0.00001
MC (166)	90	48.973	40-68	*p *< 0.00001
MC (223)	32	31.747	32-32	*p *< 0.00001
MC (292)	7	6.939	7-7	*p *< 0.00001
MC (333)	74	70.786	40-74	*p *< 0.00001
MC (336)	64	63.361	64-64	*p *< 0.00001
MC (368)	43	34.176	9-43	*p *< 0.00001
MC (697)	12	11.825	11-12	*p *< 0.00001
MC (698)	28	27.655	28-28	*p *< 0.00001

**Table 4:** Tissue compartmentalization of SRLV sequences. SRLV sequence compartmentalization between tissues within the animal (n = 7)*, using Bayesian MCMC approach for determination of Association Index (AI), Parsimony Score (PS) and Monophyletic Clade (MC) values

**Animal No**.	Statistic	No. of sequences	Mean	95% Confidence interval (CI)	Significance
166	AI	90	2.262	1.433-3.125	*p *< 0.00001
	PS	90	24.845	22-27	*p *< 0.00001
	MC (BAL)	27	9.815	5-11	*p *< 0.01
	MC (L)	12	3.260	2-4	*p *< 0.01
	MC (SC)	21	3.541	2-6	*p *< 0.05
	MC (PBMC)	20	3.411	3-5	*p *< 0.05
	MC (CPx)	10	1.773	1-3	*p *< 0.05
223	AI	32	0.381	0.209-0.602	*p *< 0.00001
	PS	32	7.896	7-9	*p *< 0.00001
	MC (CPx)	13	7.986	8-8	*p *< 0.01
	MC (L)	4	2.925	2-3	*p *< 0.01
	MC (MG)	6	2.035	2-2	*p *< 0.05
	MC (PBMC)	5	4.029	4-4	*p *< 0.01
	MC (SC)	4	1.151	1-2	Non significant
333	AI	74	1.034	0.552-1.547	*p *< 0.00001
	PS	74	15.723	14-17	*p *< 0.00001
	MC (BAL)	14	6.583	3-12	*p *< 0.01
	MC (CPx)	16	3.089	2-5	*p *< 0.05
	MC (L)	8	4.778	3-5	*p *< 0.01
	MC (MG)	6	3.977	4 -4	*p *< 0.01
	MC (PBMC)	17	4.141	3-7	*p *< 0.01
	MC (SC)	13	4.175	4-5	*p *< 0.01
336	AI	64	0.002	1.160 × 10^-9 ^- 1.953 × 10^-4^	*p *< 0.00001
	PS	64	4.204	4-5	*p *< 0.00001
	MC (BAL)	14	8.348	5-10	*p *< 0.01
	MC (L)	12	11.983	12 -12	*p *< 0.01
	MC (MG)	25	23.300	16-25	*p *< 0.01
	MC (SC)	13	10.967	11 -11	*p *< 0.01
368	AI	43	0.725	0.333-1.115	*p *< 0.00001
	PS	43	7.961	7-9	*p *< 0.00001
	MC (BAL)	14	3.872	2-7	*p *< 0.01
	MC (CPX)	15	8.632	5-11	*p *< 0.01
	MC (L)	3	1.013	1 -1	Non significant
	MC (MG)	1	1.000	1 -1	Non significant
	MC (SC)	10	8.655	6-10	*p *< 0.01
697	AI	15	0.000	0-1.878 × 10^-5^	*p *< 0.00001
	PS	15	1.000	1 -1	*p *< 0.00001
	MC (SC)	12	12.000	12 -12	*p *< 0.01
698	AI	28	0.446	0.014-0.839	*p *< 0.00001
	PS	28	5.514	4-7	*p *< 0.00001
	MC (MG)	16	4.227	2-7	*p *< 0.05
	MC (PBMC)	4	1.416	1-2	Non significant
	MC (SC)	8	6.264	6-7	*p *< 0.01

* The animal 292 was not included because less than 10 sequences were available

Overall, these results indicate strong sequence segregation between animals and the existence of tissue compartmentalization within the animal.

A signature pattern analysis was performed in search of conserved amino acid motifs specific to a particular tissue, but we were unable to find this pattern in the genetic region under analysis. Also analysis of the potential Asn-X-Ser/Thr glycosylation site was carried out [36]. This site was present in sequences from all the animals (except No. 223) and was equally distributed in different tissues, discarding glycosylation pattern as a possible explanation for compartmentalization.

## Discussion

This study provides evidence of VMV compartmentalization within the CNS and other tissues and confirms its existence in the mammary gland as previously described between blood and colostrum [32] amongst seropositive naturally infected small ruminants. The sheep had Visna-like severe lesions in the CNS, with non-suppurative meningo-encephalitis [10]. According to the specific feature of this outbreak [6,7], most animals presented lesions in the spinal cord and 50 percent in the brain. Most of them also presented lung lesions. VMV sequence distribution differed between animals, as revealed by phylogenetic analysis. The different compartmentalization patterns may be explained by differences in host genetic susceptibility, anatomical features, the genome of the infecting viruses, as well as high rates of mutation and replication, generating a quasispecies [37], and variants which may escape from surveillance [38]. Compartmentalization may involve genetic drift and founder effects [23], and/or being a consequence of different selective pressures [12].

Respiratory secretions containing infected alveolar macrophages can be responsible for SRLV transmission, especially in intensive rearing systems such as those applied in the Assaf breed of the animals under study [39]. Consequently, blood, lymph, and then target tissues, such as L, MG, CPx and SC of CNS, become infected [40]. Following purifying selection (as observed in this study) the virus evolves and compartmentalizes, producing a quasispecies. Thereafter, the virus may go back through lymphoid and blood cells to other tissues. In line with this scheme and using a novel methodology previously applied to investigate geographical origin of sequences [35], we found as expected, that intra-host ancestor sequences were not located in the CNS or other tissues, but in BAL or PBMC (when available). PBMC sequences were present in different clades, displaying the broadest variation per tissue, which is compatible with migration of virus from blood to tissues and vice versa.

However, particular ancestor/primary sequences could not be always identified. This may be due to the unavailability of PCR amplification in some samples. Thus, clones analyzed in the ancestor tissues (BAL and PBMC) may have not included the complete spectrum of ancestor viral sequences. Besides, all samples were obtained at a single time point (necropsy) even though the time points of infection by a particular virus may have not been simultaneous at different body sites. Also, there may have been re-infection events from inside or outside the body (like sheep 333, apparently with two main infections). Finally, body sites other than BAL (such as nasal and conjunctival mucosa) may have been the initial source of infection.

The virus under study belonged to the genotype A, also involved in other Visna infections [41]. Like in CNS studies of HIV [42] and in contrast with findings on FIV [14] infections, we were unable to find any CNS-specific signature pattern. In these lentiviruses, V3-V5 regions of SU encoding neutralizing epitopes may mutate, leading to viral resistance to existing antibodies also affecting cell tropism [18,30], including neurotropism and neurovirulence [14,43]. Amongst the SRLV sequences under study (TM and V4-V5), V4 - claimed to have a function analogous to that of HIV-V3 region [32,33] - was the most variable. Although variable and constant regions within the SRLV SU protein may trigger the production of neutralizing antibodies [31], the main sites/epitopes involved in this process have not been identified. Additional factors or genetic regions, such as those encoding viral proteins (Vif) that interact with host molecules (APOBEC; [44]), might be involved in neuroinvasion.

Of the 16 MG clones of animal 698, 12 showed a non-synonymous transition C to T at the same position, inserting a stop codon along the *env *region encoding the SU protein (Figure 1). The finding of stop codons along with codifying sequences suggests the emergence of quasispecies, the presence of a high rate of viral evolution and thus the existence of defective interfering particles/heteroclites. However, numerically realistic model studies suggest that these particles are unlikely to survive or influence viral dynamics [45].

Work on HIV and simian immunodeficiency virus (SIV) has shown that viral evolution in the brain is peculiar in that this immunologically privileged site allows a low level of viral replication [13] before disease onset [46], but it may be considered a "sanctuary" where the virus can evolve freely, avoiding the effects of adaptive immunity and/or therapy [12]. In line with this, the least conserved Env amino acid sequence was found in SC and the most conserved in L (data not shown). The diagnostic LTR-PCR used in this study only yielded consistent amplifications when using CNS as DNA source, since all animals were seropositive and yielded PCR-specific amplicons in CNS (Table 1). This strongly suggests that CNS is a preferred site of infection in the Visna affected animals involved. SRLV replication occurred in brain, since viral antigens p25 and gp130 were detected in CNS by immunohistochemistry and two fully replicative viral isolates, able to replicate in SCP cells, were obtained from SC and cerebrospinal fluid from two of the diseased sheep used (Nos. 697 and 698, respectively) [47]. This is compatible with studies on HIV infections, where viral replication in the CNS occurs in diseased individuals, leading then to divergent trends in different compartments [28].

This study shows that different SRLVs can coexist in the CNS from a Visna diseased sheep. This was clearly observed in animal 333, infected by two viruses (333a and 333b sequences), one closer to the remaining outbreak sequences than the other. Under the hypothesis that the strain's genetic makeup (in the SU genetic regions analyzed) and induced pathology are associated, the set of sequences most similar to other sequences in the outbreak would be causing the nervous clinical signs. The CNS 333a sub-set sequences were essentially from CPx (n = 7) and to a lower extent from SC (n = 2), whereas 333b ones were similarly represented in CPx (n = 9) and SC (n = 11). This would be consistent with a recent infection in the case of 333a, in which the virus from infected CPx cells (blood-CSF barrier) reached only a few SC sites. In the case of 333b sequences, the infection appeared to have been longer established, as it extended to various SC sites after CPx initial infection. Whether different viruses infected different cells of the same tissue (such as choroid plexus) or different viruses coinfected the same cell type remains unknown. In any case, this study demonstrates in VMV infections that, like in those by SIV, the physical continuity of the brain tissue does not necessarily result in phylogenetic proximity [13]. Disruption of the blood-brain or the blood-CSF (CPx) barriers may have taken place, as proposed in FIV and HIV infections [43,48]. In HIV infections, CPx may allow a bidirectional productive lentiviral infection of CNS and peripheral organs, having often a mixture of viral sequences [43,46]. Similarly in this study, VMV-infected cells may have crossed the CPx barrier to colonize CSF and SC, and gone back from there to blood through blood-brain or blood-CSF (CPx) barriers.

## Conclusions

The results obtained in this work provide evidence of type A SRLV compartmentalization in the CNS and other tissues. These viruses can be found in tissues that allow horizontal transmission among the animals of the outbreak. Ancestor tissue estimation strongly suggests that infection appears to start in alveolar macrophages (BAL) or blood (PBMC) when BAL was unavailable. Likely, PBMC became infected after intake of virus or infected cells/particles through respiratory and/or mammary secretions, including colostrum/milk. Moreover, the identification of sequences from mammary gland (MG) as terminal tips in the trees supports this hypothesis, indicating the two main compartments from which the virus can be transmitted.

Through this route, circulating A viruses cause neuropathological disease in the Assaf animals under study. Subsequently and according to phylogeny observations, viruses may either reach different tissues including the CNS, lung, and mammary gland and compartmentalize or be transmitted from ancestor tissues (BAL/PBMC) to new hosts.

## Methods

### Animals and diagnostic tests

This study involved seven sheep of the Assaf breed - six females (223, 292, 333, 336, 697 and 698) and one male (166) - and a Milchschaf breed ewe (368), ranging in age from 2 to 5 years. Sheep were from different dairy farms (except for 333 and 336, which were from the same flock) located in the Autonomous Community of Castilla-y-León (North-Western Spain), where several cases of Visna Maedi nervous form had been previously reported [9]. All sheep showed clinical nervous symptoms and post-mortem analysis was consistent with Visna lesions (Table 1).

Animal handling, euthanasia and experimental procedures were carried out in compliance with the current European and national (RD 1201/2005) regulations, with the approval of the Comité de Ética y Experimentación Animal of the University of León and authorization of the Gobierno de Castilla y León. Euthanasia was done by intravenous injection of barbiturate overdose followed by exsanguination. Complete necropsy analysis was performed in the animals. Animals were submitted to necropsy and used for clinical and pathology studies at the University of León following the farmers' request and consent.

For serological and molecular diagnosis of SRLV infection, animals were analyzed serologically using a commercially available ELISA (Elitest-MVV^®^; HYPHEN Biomed, France); and by PCR on viral promoter LTR (about 300 bp), using primers and a procedure previously described [49] for proviral DNA amplification.

### Samples

Whole blood from each animal was collected to isolate plasma for ELISA and peripheral blood mononuclear cells (PBMC) for PCR analysis. These were isolated from EDTA-blood samples by Ficoll-Hypaque gradient centrifugation (d = 1.077; Lymphoprep Axis-Shield, Oslo). One cubic centimetre samples of spinal cord (SC), choroid plexus (CPx), mammary gland (MG), lung (L), and bronchoalveolar lavage (BAL) were also collected post-mortem and embedded in RNAlater (Qiagen) until use. Samples from brain, SC, L and MG were also obtained and fixed in buffered formalin 10% for histopathological studies. Genomic DNA was extracted from PBMC, BAL and tissue samples with QIAamp^® ^DNA Blood Mini Kit (Qiagen), following the manufacturer's instructions. Tissue samples were previously lysed in buffer (100 mM Tris-HCl pH 8.5; 5 mM EDTA; 400 mM NaCl; 0.2% SDS) containing Proteinase K (50 μg/ml per 10^7 ^PBMC, Sigma) at 56°C for 1 h.

### PCRs and cloning for compartmentalization

To avoid template resampling [50], DNA was diluted in fivefold series and amplified by PCR in triplicate (limiting-dilution PCRs). Diluted DNA was used in a seminested PCR that was carried out with primers previously described [51] slightly modified for this study. Briefly, the reaction mix of the first round of seminested PCR consisted in: Buffer Certamp 1 × (Biotools), 2 mM MgCl_2 _(Biotools), 225 μM of each dNTP (Biotools), 600 nM of each primer, 0.04 U/μl of Certamp enzyme (Biotools) and sample DNA diluted 5-fold serially in a final volume of 50 μl. PCR conditions were: initial denaturation step for 5 min at 95°C followed by 45 cycles of 95°C for 60 s, annealing at 47°C for 60 s and extension at 72°C for 60 s, followed by a final extension step of 10 min at 72°C. Primers were: #563 FW 5'-GAYATGRYRGARCAYATGACA-3' (7272-7292 nt); #564 RV 5'-GCYAYRTGCTGYACCATGGCATA-3' (8089-8067 nt). For the second round, 5 μl of the first PCR were transferred to the new reaction mix. Reaction conditions for the second round of PCR were identical to those described for the first round, with the exception that the #567 FW primer (#567 FW 5'-GGGNACNARIACRAAYTGGAC-3'; 7481-7501 nt) was used and the annealing temperature was increased to 49°C. A 607 bp amplicon was obtained encompassing the C-terminal part of surface protein (SU) including V4, C4 and V5 regions and the N-terminal part of transmembrane (TM) protein encoded by the *env *gene (7481-8089 nt of CAEV CO, M33677). Amplified products were chilled at 4°C. A negative control reaction without template DNA was included for each primer pair to control potential contaminations. In order to minimize the risk of contamination, mixing of reagents, sample preparation, and analysis of the amplified products were performed in three separate areas.

PCR amplicons were resolved on 1.5% agarose gels, and bands of the expected sizes were excised and purified with QIAquick Gel Extraction Kit (Qiagen), and subsequently cloned into the vector pGEMT-easy^® ^(Promega) following the manufacturer's instructions. Fifty to 100 transformed colonies (*Escherichia coli *XL1-Blue) were picked per sample and grown overnight at 37°C in LB medium (1% Bacto Tryptone, 0.5% yeast extract, 1% NaCl) with 100 μg/ml ampicillin. After selection of positive clones upon screening by restriction enzyme digestion (EcoRI), plasmid DNA was purified from bacterial culture using the Quantum Prep^® ^Plasmid Miniprep kit (Bio-Rad) according to the manufacturer's instructions. Purified plasmid DNA was sequenced using an ABI PRISM 310 Genetic Analyzer (Applied Biosystems).

### Sequence analysis

The total number of sequences obtained in the group of 8 animals was 350: 54 from CPx, 88 from SC, 39 from L, 46 from PBMC, 69 from BAL and 54 from MG.

Nucleotide sequences were analyzed with Chromas 2.23 (Technelysium, Helensvale, Australia) and edited using BioEdit software [52]. The corresponding amino acid sequences were obtained according to the Expasy server. Multiple alignments for comparison and phylogenetic studies were made with the ClustalW [53] respecting the reading frame. The nucleotide distance matrix was calculated with the PAUP* software [54]. The presence of natural selection was evaluated as the estimation of ω, the ratio between the number of non-synonymous substitutions per non-synonymous site (dN) and the number of synonymous substitutions per synonymous site (dS). The ω estimation was carried out using Single-Likelihood Ancestor Counting (SLAC, [55]) method implemented in Datamonkey webserver [56].

A phylogenetic tree was created evaluating the best model of molecular evolution (ModelTest software; [57]) and using Bayesian heuristic approaches (BEAST (16) and MrBayes software; [58]).

To explore the overall compartmentalization in the sequence data we employed a Bayesian Markov chain Monte Carlo (MCMC) approach (program BaTS; [59]). This analysis was based on the trees output from the MrBayes BEAST analysis described above, with 10% removed as burn-in and employing 1000 replications. From these trees we computed the significance of the parsimony score (PS) and association index (AI) statistics of the strength of geographical clustering by phylogeny [59]. In addition, we computed the monophyletic clade (MC) statistic that measures the strength of clustering within individual geographic regions. Thus, common ancestors were not inferred from the tree topology directly, since tree clusters were based only on minimal differences between the sequences compared (independently of the pathways used in diversification) whereas the ancestor determination applied was a complex process, determining the most parsimonious pathway to maximally relate the set of sequences under study while minimizing the phyletic diversity. Importantly, the common ancestor approach accounts for uncertainty in the underlying phylogeny by using a large number of plausible trees. In order to understand correctly the results, we have considered that low AI and PS values represent strong phylogeny-trait association and MC is positively correlated with the strength of the phylogeny-trait association [59]. Thus, high AIs and PSs and low MCs show sequences' compartmentalization. To better understand the clonal origin of collected sequences, Bayesian MCMC approaches described by Lemey and coauthors [35] and developed for phylogeographical applications were applied. Specifically, according to Lemey and collaborators, the *state *of each sequence was represented by its geographical origin and the analyses lead to infer about the *state *of each internal node: this approach allowed to follow the viral evolution through the *space *of assigned *states*. We used BEAST package software [60] to infer about the *state *of internal nodes within the phylogeny of each animal. In this work, we assigned as *state *the animal tissue from which each sequence was obtained. Parameters were estimated using the Bayesian method implemented in the BEAST software package and the convergence of chains was assessed by calculating the Effective Sample Size (ESS) of the runs. All parameter estimates showed significant ESS (> 200). Maximum Clade Credibility (MCC) phylogeny and information about common ancestors' *state *were obtained using Tree Annotator.

A signature pattern analysis was carried out to search for possible amino acid positions that would provide a conserved pattern within the SNC Env sequences relative to the PBMC or BAL Env sequences. The analysis was calculated with the VESPA software [61] that determines the frequency of an amino acid at a specific position and then determined whether there was a distinct pattern for one set of sequences.

### Nucleotide sequences accession number

The SRLV-*env *sequence data generated in this study were deposited into GenBank database and are available under accession numbers: BAL 166 (JF288187-JF288213), Cpx 166 (JF288214-JF288223), L 166 (JF288224-JF288235), PBMC 166 (JF288236-JF288255), SC 166 (JF288256-JF288276), Cpx 223 (JF288277-JF288289), L 223 (JF288290-JF288293), MG 223 (JF288294-JF288299), PBMC 223 (JF288300-JF288304), SC 223 (JF288305-JF288308), SC 292 (JF288309-JF288315), BAL 333 (JF28831-JF288329), Cpx 333 (JF288330-JF288345), L 333 (JF288346-JF288353), MG 333 (JF288354-JF288359), PBMC 333 (JF288360-JF288376), SC 333 (JF288377-JF288389), BAL 336 (JF288390-JF288403), L 336 (JF288404-JF288415), MG 336 (JF288416-JF288440), SC 336 (JF288441-JF288453), BAL 368 (JF288454-JF288467), Cpx 368 (JF288468-JF288482), L 368 (JF288483-JF288485), MG 368 (JF288486), SC 368 (JF288487-JF288496), SC 697 (JF288497-JF288508), MG 698 (JF288509-JF288524), PBMC 698 (JF288525-JF288528), SC 698 (JF288529-JF288536).

## Competing interests

The authors declare that they have no personal, financial or non financial competing interests.

## Authors' contributions

HR, AC, M-MH, BS-R, IG, XA, HC and PJ carried out the immunoassays, molecular genetic studies and participated in the sequence alignment. LB performed the statistical analysis and helped to draft the manuscript. JB, LP, VP and J-F G-M carried out the histopathological study. HR, RA, BA and DA conceived of the study, and participated in its design and coordination and helped to draft the manuscript. All authors read and approved the final manuscript.

## Supplementary Material

Additional file 1 **Phylogenetic tree including 350 partial env sequences from 8 animals**. Taxa names include the animal number, the tissue from which the sequence was obtained and the clone number. Tissues are identified by a three-letter code as follows: broncho-alveolar macrophages BAL, choroid plexus CPx, spinal cord SCO, peripheral blood mononuclear cells PBM, mammary gland MGL, lung LUN. Animal clusters are depicted in different colors. Sequences presenting stop codons within the 698 animal clade are reported in gray. Posterior probability supporting tree's nodes are reported. Click here for file
