# Supplementary material for: Study of compartmentalization in the visna clinical form of small ruminant lentivirus infection in sheep
Source: BMC Vet Res. 2012 Jan 26;8:8. doi: 10.1186/1746-6148-8-8 (PMC3328241; doi:10.1186/1746-6148-8-8)
Supplement: Additional file 1 — Phylogenetic tree including 350 partial env sequences from 8 animals. Taxa names include the animal number, the tissue from which the sequence was obtained and the clone number. Tissues are identified by a three-letter code as follows: broncho-alveolar macrophages BAL, choroid plexus CPx, spinal cord SCO, peripheral blood mononuclear cells PBM, mammary gland MGL, lung LUN. Animal clusters are depicted in different colors. Sequences presenting stop codons within the 698 animal clade are reported in gray. Posterior probability supporting tree's nodes are reported. [file 1746-6148-8-8-S1.PDF]

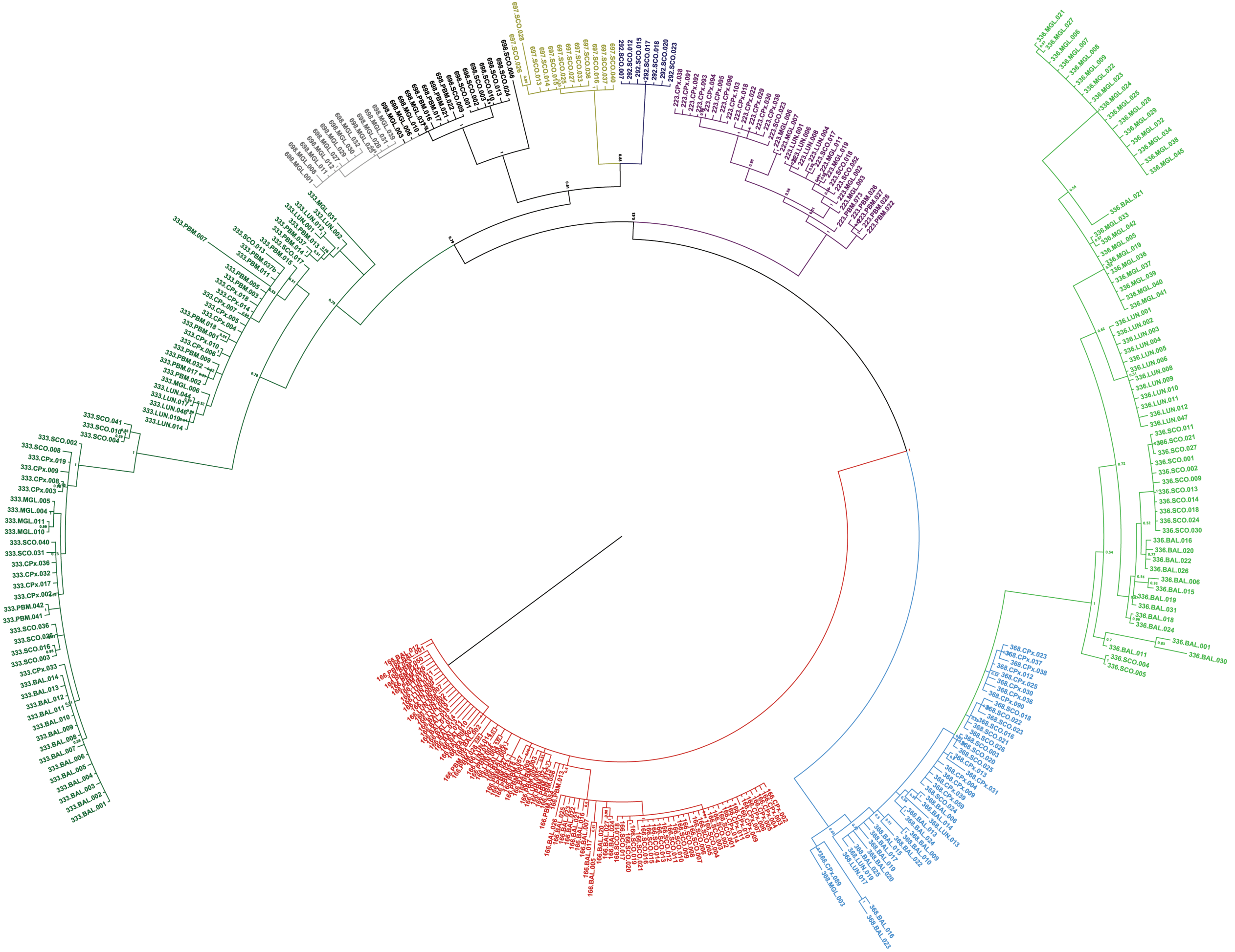
336.MGL.021
336.MGL.027
336.MGL.006
336.MGL.007
336.MGL.008
336.MGL.009
336.MGL.022
336.MGL.023
336.MGL.024
336.MGL.025
336.MGL.028
336.MGL.029
336.MGL.032
336.MGL.034
336.MGL.038
336.MGL.045
336.BAL.021
336.MGL.033
336.MGL.042
336.MGL.005
336.MGL.019
336.MGL.036
336.MGL.037
336.MGL.039
336.MGL.040
336.MGL.041
336.LUN.001
336.LUN.002
336.LUN.003
336.LUN.004
336.LUN.005
336.LUN.006
336.LUN.008
336.LUN.009
336.LUN.010
336.LUN.011
336.LUN.012
336.LUN.047
336.SCO.011
336.SCO.021
336.SCO.027
336.SCO.001
336.SCO.002
336.SCO.009
336.SCO.013
336.SCO.014
336.SCO.018
336.SCO.024
336.SCO.030
336.BAL.016
336.BAL.020
336.BAL.022
336.BAL.026
336.BAL.006
336.BAL.015
336.BAL.019
336.BAL.031
336.BAL.018
336.BAL.024
336.BAL.001
336.BAL.030
336.BAL.011
336.SCO.004
336.SCO.005
333.SCO.041
333.SCO.010
333.SCO.004
333.SCO.002
333.SCO.008
333.CPx.019
333.CPx.009
333.CPx.008
333.CPx.003
333.MGL.005
333.MGL.004
333.MGL.011
333.MGL.010
333.SCO.040
333.SCO.031
333.CPx.036
333.CPx.032
333.CPx.017
333.CPx.002
333.PBM.042
333.PBM.041
333.SCO.036
333.SCO.026
333.SCO.016
333.SCO.003
333.CPx.033
333.BAL.014
333.BAL.013
333.BAL.012
333.BAL.011
333.BAL.010
333.BAL.009
333.BAL.008
333.BAL.007
333.BAL.006
333.BAL.005
333.BAL.004
333.BAL.003
333.BAL.002
333.BAL.001
333.PBM.007
0.3
